# Effects of glutamate and aspartate on prostate cancer and breast cancer: a Mendelian randomization study

**DOI:** 10.1186/s12864-022-08442-7

**Published:** 2022-03-16

**Authors:** Yindan Lin, Ze Yang, Jingjia Li, Yandi Sun, Xueyun Zhang, Zihao Qu, Yan Luo, Lihong Zhang

**Affiliations:** 1 Department of Biochemistry and Cancer Institute, (Key Laboratory of Cancer Prevention and Intervention of China National MOE), Zhejiang University School of Medicine, No. 866 Yuhangtang Road, Xihu District, Zijingang Campus, Zhejiang 310058 Hangzhou, China; 2 Orthopedic Research Institute of Zhejiang University, Zhejiang 310058 Hangzhou, China

**Keywords:** Glutamate, Aspartate, Prostate cancer, Breast cancer, Mendelian randomization

## Abstract

**Background:**

Respectively, prostate cancer (PCa) and breast cancer (BC) are the second most and most commonly diagnosed cancer in men and women, and they account for a majority of cancer-related deaths world-wide. Cancer cells typically exhibit much-facilitated growth that necessitates upregulated glycolysis and augmented amino acid metabolism, that of glutamine and aspartate in particular, which is tightly coupled with an increased flux of the tricarboxylic acid (TCA) cycle. Epidemiological studies have exploited metabolomics to explore the etiology and found potentially effective biomarkers for early detection or progression of prostate and breast cancers. However, large randomized controlled trials (RCTs) to establish causal associations between amino acid metabolism and prostate and breast cancers have not been reported.

**Objective:**

Utilizing two-sample Mendelian randomization (MR), we aimed to estimate how genetically predicted glutamate and aspartate levels could impact upon prostate and breast cancers development.

**Methods:**

Single nucleotide polymorphisms (SNPs) as instrumental variables (IVs), associated with the serum levels of glutamate and aspartate were extracted from the publicly available genome-wide association studies (GWASs), which were conducted to associate genetic variations with blood metabolite levels using comprehensive metabolite profiling in 1,960 adults; and the glutamate and aspartate we have chosen were two of 644 metabolites. The summary statistics for the largest and latest GWAS datasets for prostate cancer (61,106 controls and 79,148 cases) were from the Prostate Cancer Association Group to Investigate Cancer Associated Alterations in the Genome (PRACTICAL) consortium, and datasets for breast cancer (113,789 controls and 133,384 cases) were from Breast Cancer Association Consortium (BCAC). The study was performed through two-sample MR method.

**Results:**

Causal estimates were expressed as odds ratios (OR) and 95% confidence interval (CI) per standard deviation increment in serum level of aspartate or glutamate. Aspartate was positively associated with prostate cancer (Effect = 1.043; 95% confidence interval, 1.003 to 1.084; *P* = 0.034) and breast cancer (Effect = 1.033; 95% confidence interval, 1.004 to 1.063; *P* = 0.028); however, glutamate was neither associated with prostate cancer nor with breast cancer. The potential causal associations were robust to the sensitivity analysis.

**Conclusions:**

Our study found that the level of serum aspartate could serve as a risk factor that contributed to the development of prostate and breast cancers. Efforts on a detailed description of the underlying biochemical mechanisms would be extremely valuable in early assessment and/or diagnosis, and strategizing clinical intervention, of both cancers.

**Supplementary Information:**

The online version contains supplementary material available at 10.1186/s12864-022-08442-7.

## Introduction

The prostate (man) and breast cancers (woman) are almost the most frequently diagnosed cancers that also constitute a major cause of cancer-related deaths [1, 2]. In the western countries, prostate cancer is the most common form of cancer among men of 50yrs and older with a mortality-to-incidence ratio of 20% [3]. As for breast cancer, the United States alone in 2017 recorded 255,180 new cases and 41,070 deaths [4]. Thus, despite tremendous advancements over the previous decade, early detection/treatment of prostate and breast cancers is next to satisfaction.

In cells, nutrients are essential for energetics and many types of bio-mass building. Accumulating evidence from basic studies suggests that cancer cells continuously adapt to dynamic metabolic micro-environment by changing the way of nutrient utilization during malignancy development. For instance, under aerobic condition, cancer cells often exhibit upregulated glycolysis for their rapid growth [5]; and they also use amino acids, in particular glutamine and aspartate, as anaplerotic nutrients for TCA cycle that is coupled with oxidative phosphorylation [6]. Hence, cancer cells are generally vulnerable to nutrient deficiency, a feature that potentially provides new targets for cancer therapy [7]. Indeed, given altered metabolism typical of cancer tissues [8], quite an ever-growing number of epidemiological studies have exploited metabolomics to research the etiology and figure out biomarkers for early detection, or progression of prostate cancer [9].

Observationally, soy proteins, rich in glutamate and aspartate, are reported to lower the androgen levels but no large RCTs have been conducted to test their health effects; in addition, animal experiment results suggest that glutamate and aspartate can decrease the testosterone levels [10, 11], and diverse epidemiological studies suggest that consumption of soy, fruits, and vegetables are linked with reduced risk of recurrence and increased survival rate of prostate cancer and breast cancer [12–15]. In a RCT of men, _D_-aspartate can reduce testosterone [16]. Despite these studies, however, up to now a causal relationship between serum levels of amino acids, such as glutamate and aspartate, and prostate and breast cancers remains elusive. Furthermore, at times the metabolic studies generate results that are not always consistent due to the differences in outcome examined, metabolomics platforms exploited, and characteristics and/or sizes of study populations.

MR, as a newly approach, gets information from genome-wide association studies (GWAS) to evaluate the causal relationship between exposures and phenotype without any potentially harmful intervention [17, 18]. Briefly, MR analysis widely utilizes the power of genetic variants as IVs to evaluate the causal associations between risk factors and disease outcomes [19]. Because genetic variants are inherently inherited at conception, an MR analysis can avoid potential bias along with misinterpretation of results by removing confounding factors in traditional observational studies typically associated with socio-economic status, lifestyle (alcohol and smoking) and health status. Mendel’s second law dictates that each pair of alleles undergoes independent assortment without interference from environmental factors. Two-sample MR analysis requires summary-level data from two independent GWASs for putative exposures and outcomes [20]. Here, exploiting genetically instrumented glutamate and aspartate from GWAS [21] and large case–control studies of prostate and breast cancers with extensive genotyping [22, 23], an MR study was performed to estimate the causal effects of serum glutamate and aspartate levels and the development of prostate and breast cancers.

## Subjects and methods

### Study design and data sources

**Fig. 1:**
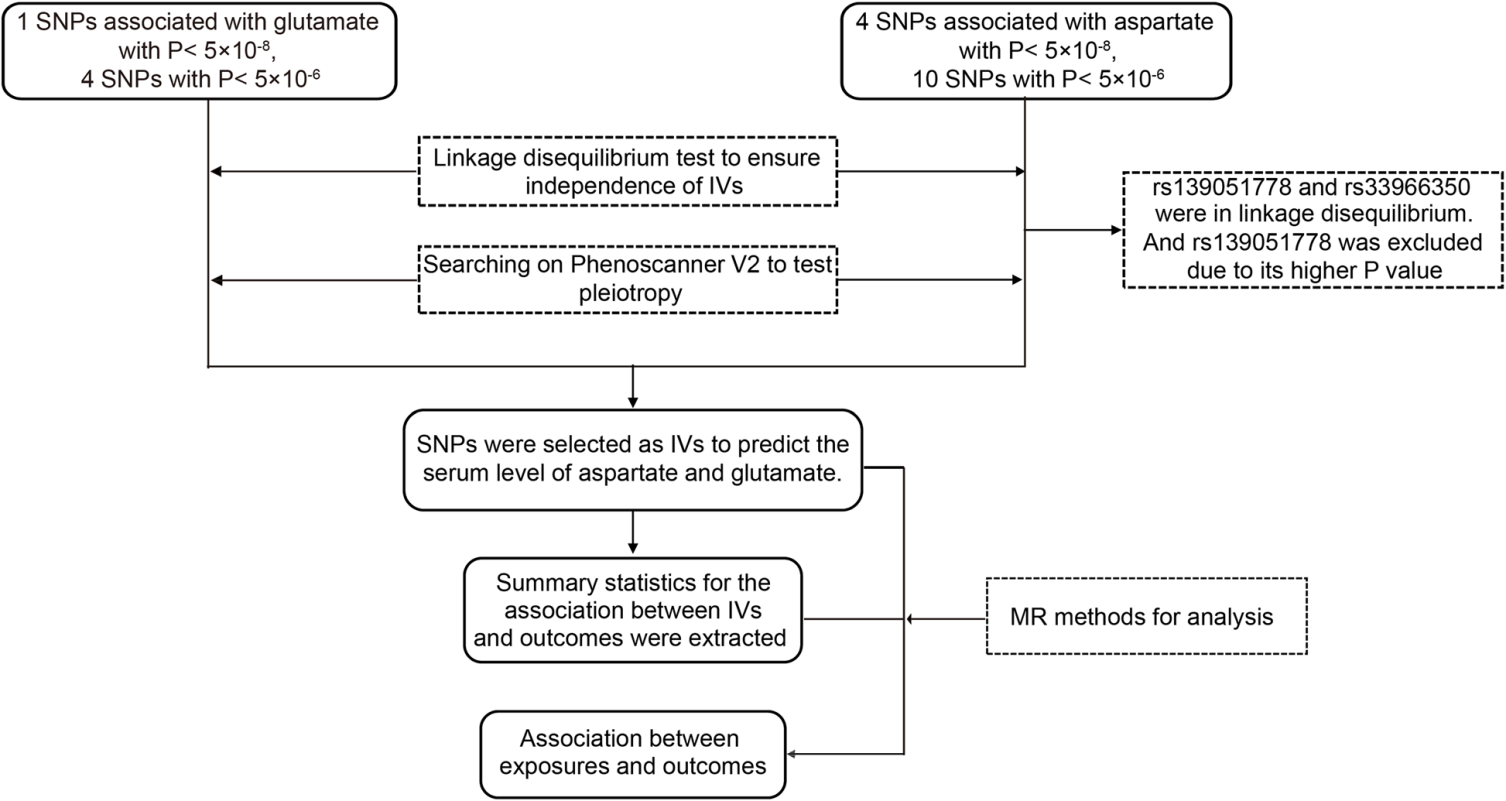
The flowchart of Mendelian Randomization analysis of serum levels of glutamate and aspartate and risk of development of breast cancer and prostate cancer

As shown in Fig. 1, a two-sample Mendelian Randomization approach was designed in this study. It is based on the assumption that instrumental variables are related to serum levels of glutamate and aspartate, but independent of the risk of cancer and cofounders.

The IVs were extracted from the publicly available genome-wide association studies, which were a common, low-frequency and rare variants GWASs, and conducted in 1,960 adults to associate genetic variations with blood metabolites by comprehensive metabolite profiling [21]. The glutamate and aspartate chosen in our study were two of the whole 644 metabolites according three longitudinal data collections. SNPs were removed as the call rate was less than 95%, the *P* value was above 10^−6^ and the minor allele frequency was less than 1%. Information of data on the association of SNPs with serum glutamate and aspartate and the association of SNPs with breast cancer and prostate cancer were obtained from the GWAS database [22, 23].

### Genetic instruments for glutamate and aspartate

Genetic signatures, such as SNPs, associated with glutamate or aspartate were obtained from a large GWAS study [21], in which the participants were of European origin. SNPs as IVs, were not confounded by socio-economic status and lifestyle factors (alcohol and smoking). Different genetic variants were obtained with different cutoffs for significance, genome-wide association significance (5 × 10^−8^) or a less stringent significance (5 × 10^−6^). The strength of each SNP was evaluated using the F-statistic, calculated using a well-established formula [24]. A cutoff of 10 as a "rule of thumb" was used to distinguish between strong and weak instruments [25]. Weak instruments can bias the findings. An MR approach is based on the hypothesis that genetic exposure influences the outcome directly [26], otherwise, it is not suitable to perform the analysis in the presence of pleiotropy. Thus, to validate that the SNPs were associated with prostate or breast cancer solely via glutamate or aspartate, pleiotropy was checked, i.e., genetic associations with prostate or breast cancer via estrogen-related factors such as breast density and use of hormone replacement therapy [27, 28] and high body mass index (BMI) [29], with PhenoScanner V2 website (www.phenoscanner.medschl.cam.ac.uk). It is a comprehensively curated genetic cross-reference system and provides all well-established associations of known SNPs with their phenotypes, including subgenome-wide associations [30]. In order to make sure the independent contribution of selected SNPs, i.e., the correlation between the selected SNPs, LD-link website (https://ldlink.nci.nih.gov/, population: CEU) was used to perform a linkage disequilibrium (LD) test, which was a suite of web-based applications designed to easily and efficiently interrogate LD in population groups. For SNPs (*r*^2^ < 0.8), the SNPs (5 × 10^−8^) were used. For less strongly associated SNPs (5 × 10^−6^), only the uncorrelated SNPs (*r*^2^ < 0.01) were chosen.

### Genetic associations with prostate and breast cancers

Summary data for prostate cancer was extracted from the largest GWAS meta-analysis including 79,148 cases and 61,106 controls of European ancestry from the PRACTICAL consortium (http://practical.icr.ac.uk/blog/) [23]. Summary statistics for breast cancer was extracted from the latest and largest meta-GWAS from BCAC including 133,384 cases and 113,789 controls (http://bcac.ccge.medschl.cam.ac.uk/) [22]. The participants were women of European ancestry. The written consent of listed participants was provided, and all the studies from which we extracted data for our MR analysis were supported by the ethical review boards.

### Statistical analysis

In order to evaluate the casual effect between glutamine and aspartate and cancers, MR methods including simple median, weighted median (WM), penalized weighted median, the inverse-variance-weighted (IVW), penalized IVW, robust IVW, penalized robust IVW, MR-Egger, penalized MR-Egger, robust MR-Egger, and penalized robust MR Egger are selected for analysis. Penalized analyses would give consistent estimates if a plurality of the instrumental variables are valid [31–33]. Among them, IVW is the major analyses method. For multiple independent genetic variants, IVW could weigh the average of these single causal estimates using the inverse of their approximate variances as weights [34]. The weighted median method [35] and MR Egger [34] were conducted in the sensitivity analysis to account for potential bias from unknown pleiotropy. The WM method could be considered to account for differences in the precision of estimates and could provide consistent estimates even if 50% of the information comes from invalid SNPs [35]. The simple median estimator is calculated as the median of the Wald ratio estimates [ratio of SNP on outcome to SNP on glutamate and aspartate]. The estimate obtained by WM method were validated as IVs were greater than or equal to three SNPs [36]. Otherwise, the Wald ratio was directly used as there was one SNP. Causal estimate was also obtained from MR-Egger, which was based on the assumption that the pleiotropic effects were independently distributed from the genetic associations with the exposure [34]. The intercept from MR Egger was checked whether it was nonzero because this indicates that some of the genetic predictors might be acting other than via the exposure (i.e., directionally pleiotropic) [34], invalidating the IVW estimates. The MR Egger estimate is less precise than that from IVW, because the variance of the MR Egger estimate additionally depends on the variability between the genetic associations with the exposure, and it is much larger than that from IVW [34]. Supplementing these more widely used approaches, a robust adjusted profile score (RAPS), that is robust to idiosyncratic pleiotropy [37], and improved MR methods including the robust option, penalized option, and the penalized option of the weighted median, IVW, and MR-Egger [38] were recently developed MR methods.

Heterogeneity test was performed using Cochran’s Q-test to identify whether the MR results were biased by the potential heterogenic factors. A leave-one-out permutation test was performed to assess whether the IVW estimate was biased by the influence of particular SNPs. Causal estimates between glutamate and aspartate levels and prostate and breast cancers risk were expressed as odds ratios (OR) and 95% confidence interval (CI) per standard deviation increment in plasma glutamate or aspartate level.

All the analyses with *P* < 0.05 were considered statistically significant. All statistical analyses were performed using the R Studio (R version 4.0.2) software and the R package “Mendelian Randomization”.

## Results

### Genetic instruments for glutamate and aspartate

The SNPs as the potential IVs obtained from the large GWASs of European ancestry [21], were not confounded by socio-economic status and lifestyle factors (alcohol and smoking). By using different cutoffs for significance, the different genetic variants were selected. With genome-wide association significance (*P* < 5 × 10^−8^), 5 SNPs were selected, including 1 SNP for glutamate and 4 SNPs for aspartate. However, the result of the linkage disequilibrium test on the LD-link website showed that rs139051778 and rs33966350 associated with aspartate were not independent. In fact, these two SNPs were within a same gene; thus, to get more reliable results, rs139051778 were excluded for analysis, as rs33966350 were with lower *P* value. With a less stringent significance (*P* < 5 × 10^−6^), 14 genetic variants were associated with serum glutamate or aspartate levels. Finally, the characteristics of these 18 genetic variants in total were shown in Table 1.

**Table 1:** Characteristics of the instrumental variables for glutamate and aspartate and the causal associations bewteen glutamate and aspartate with prostate and breast cancers

Exposure	SNPs	Gene^a^	Chromosome	Effect allele	Association with exposure					Association with prostate cancer			Association with breast cancer		
					Beta^b^	SE^c^	*P* value	*r*^*2*^	F	Beta^b^	SE^c^	*P* value	Beta^b^	SE^c^	*P* value
Glutamate	SNP with genome-wide significance					—				—	—	—	—	—	—
	rs239614	*LOC102724355*	21	G	0.28	0.06	4.70E-08	0.0111	22	0.0080	0.0116	0.4895	0.0115	0.0087	0.1857
	Additional SNPs with *P *< 5 × 10^−6^														
	rs261076	*DOCK2*	5	A	-0.16	0.05	1.90E-06	0.0051	10	0.0091	0.0100	0.3579	0.0105	0.0075	0.1584
	rs495578	*PDZD2*	5	A	-0.17	0.04	3.30E-06	0.0091	18	0.0047	0.0105	0.6570	0.0056	0.0080	0.4845
	rs41308216	*PIKFYVE*	2	G	-0.85	0.16	4.20E-06	0.0141	28	0.0114	0.0304	0.7077	-0.0294	0.0237	0.2149
	rs10973935	*ANKRD18A*	9	G	-0.35	0.09	4.30E-06	0.0076	15	-0.0054	0.0184	0.7701	0.0024	0.0139	0.8623
Aspartate	SNP with genome-wide significance														
	rs33966350	*ENPEP*	4	A	-1.5	0.19	1.40E-17	0.0307	62	-0.0425	0.0375	0.2565	-0.0443	0.0272	0.1031
	rs113141482	*GPR158*	10	A	-0.98	0.19	1.00E-09	0.0136	27	-0.1032	0.0497	0.0379	-0.0185	0.0377	0.6233
	rs189080637	*—*	3	A	-0.98	0.21	4.30E-08	0.0111	22	-0.0370	0.0420	0.3779	-0.0488	0.0315	0.1214
	Additional SNPs with *P* < 5 × 10^−6^														
	rs113367140	*RTP4*	3	A	-0.54	0.13	7.30E-08	0.0086	17	-0.0179	0.0229	0.4348	0.0113	0.0176	0.5195
	rs6894601	*—*	5	G	-0.16	0.04	1.90E-06	0.0081	16	-0.0057	0.0088	0.5158	0.0083	0.0066	0.2078
	rs114190931	*JARID2*	6	T	0.68	0.14	2.10E-06	0.0121	24	-0.0077	0.0241	0.7505	0.0380	0.0187	0.0425
	rs76197627	*MGRN1*	16	T	-0.49	0.13	2.20E-06	0.0071	14	-0.0315	0.0289	0.2754	-0.0038	0.0224	0.8634
	rs117109641	*APBA2*	15	T	0.65	0.17	2.40E-06	0.0076	15	0.0084	0.0321	0.7931	-0.0079	0.0240	0.7421
	rs35355649	*MORN1*	1	T	-0.17	0.05	2.60E-06	0.0061	12	0.0051	0.0097	0.6001	0.0010	0.0072	0.8932
	rs57037252	*ZNF559*	19	T	-0.36	0.08	3.10E-06	0.0101	20	0.0088	0.0151	0.5596	-0.0032	0.0113	0.7742
	rs79447732	*DLGAP2*	8	G	-0.54	0.14	3.40E-06	0.0076	15	0.0223	0.0269	0.4065	-0.0101	0.0200	0.6113
	rs137948393	*SLC4A5*	2	G	0.41	0.13	3.90E-06	0.0051	10	0.0673	0.0341	0.0487	0.0086	0.0260	0.7401
	rs75527467	*ITPR1*	3	T	1	0.3	4.10E-06	0.0056	11	0.0822	0.0389	0.0348	-0.0142	0.0365	0.6974

^a ^*DOCK2* Dedicator of Cytokinesis 2, *PDZD2* PDZ Domain containing 2, *PIKFYVE* Phosphoinositide Kinase, FYVE-type zinc finger containing, *ANKRD18A* Ankyrin Repeat Domain 18A, *ENPEP* glutamyl arninopeptidase, *GPR158* G Protein-coupled Receptor 158, *RTP4* Receptor Transporter Protein 4, *JARID2* Jumonji and AT-rich Interaction Ddomain containing 2, *MGRN1* Mahogunin Ring finger 1, *APBA2* Amyloid Beta precursor Protein Binding family A member 2, *ZNF559* Zinc Finger Protein 559, *DLGAP2* DLG Associated Protein 2, *SLC4A5* Solute Carrier family 4 member 5, *ITPR1* Inositol 1,4,5-trisphosphate receptor type 1

^b ^Beta, per allele effect on exposure or outcomes

^c ^*SE* Standard Error

The selected SNPs were from genes thought to be functionally relevant to the exposures and none of the SNPs were associated with key confounders. The SNP rs113141482 is on the gene *GPR158*, which encodes G protein-coupled receptor 158 (GPR158). GPR158 is a newly characterized cell surface protein that plays the same role, as other G-protein coupled receptors (GPCRs), on promoting prostate cancer (PCa) malignancy. Indeed, currently, the glutamate family member GPR158 is a therapeutic target for PCa [39]. The SNP rs33966350 is a locus on the gene *ENPEP* associated with blood pressure [35] and is related to aspartate, given that the gene is also relevant to the metabolism of aspartate in function: *ENPEP* encodes glutamyl aminopeptidase, catalyzing the cleavage of glutamate and aspartate from the N-terminal polypeptides.

### Associations with prostate and breast cancers

**Fig. 2:**
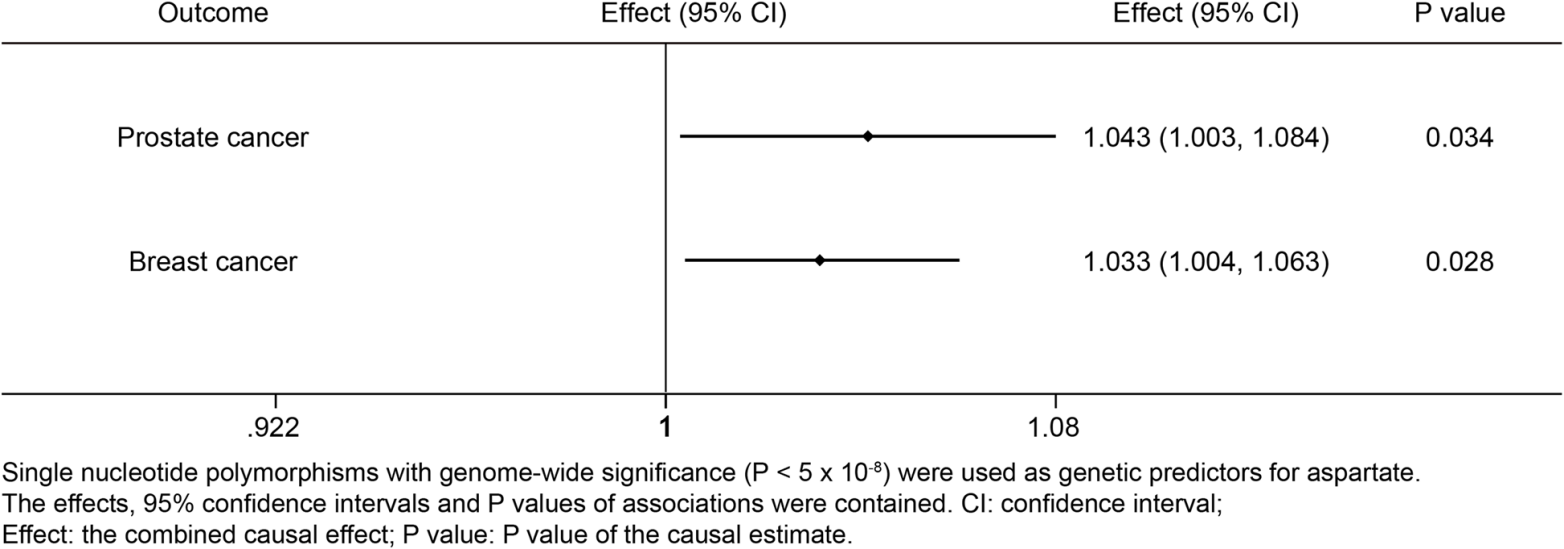
Causal associations between aspartate and prostate and breast cancers

Based on the single genome-wide significant SNPs, the result of IVW analysis (Figs. 2 and S1) was shown that the genetically instrumented aspartate was positively associated with prostate and breast cancers. Importantly, when less strongly associated SNPs was included, the results from the improved the MR methods including the robust IVW and penalized robust IVW methods also showed a significant association with prostate cancer (Table S1) and breast cancer (Table S2). Interestingly, the intercept of MR Egger, penalized MR-Egger, robust MR-Egger and penalized robust MR-Egger were not equal to zero and there was no pleiotropy. In addition, the *P* values of MR Egger, penalized MR-Egger, robust MR-Egger and penalized robust MR-Egger were less than 0.05. Therefore, the causal association in the Figs. 2 and S1 was further supported. However, the association between aspartate and breast cancer did not remain in the result of IVW analysis when including less strongly associated SNPs (Figs. S2 and S4).

**Fig. 3:**
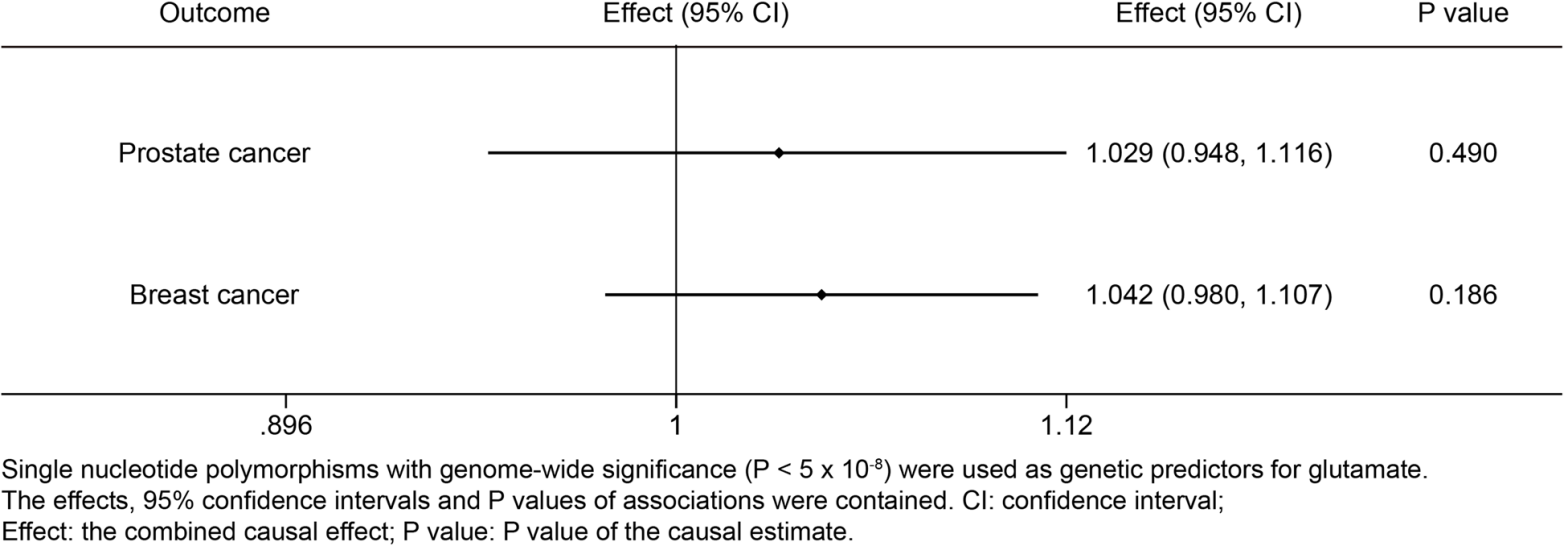
Causal associations between glutamate and prostate and breast cancers

Genetically instrumented glutamate was not significantly associated with breast cancer and prostate cancer based on the single genome-wide significant SNPs (Fig. 3). These associations were generally robust to different SNP selections as less strongly associated SNPs were included (Figs. S3, S5, Tables S3 and S4).

The summary information of GWASs on outcomes was displayed in Table S5. And the genetic associations between serum levels of glutamate and aspartate and the outcomes were shown in Table 1.

### Sensitivity analysis

As IVs were greater than or equal to three SNPs, the MR-Egger intercept test was performed to examine the pleiotropy. The intercept values from MR Egger analysis were nonzero, suggesting that the MR Egger estimate may have greater validity and the pleiotropy did not bias the results.

The single genome-wide significant SNPs in the Tables 2 and 3 and less strongly associated SNPs in the Tables S1 and S2 were to predict serum level of aspartate and the result of MR-Egger intercept test indicated that the intercept of the MR-Egger did not significantly differ from zero. Therefore, no signs of directional pleiotropy among these instruments were discovered. Less strongly associated SNPs in Tables S3 and S4 were to predict serum level of glutamate and the similar result of MR-Egger intercept test was shown. There was only one SNP at genome-wide association significance to predict glutamate, the MR-Egger intercept test could not be performed (Table 4). Additionally, robust adjusted profile score (RAPS) was also used to test the idiosyncratic pleiotropy, which further confirm the IVW findings (Tables 2, 3, 4 and Tables S1, S2, S3, S4). The single MR estimates were provided from each of the genetic variants using MR-Egger method (Fig. S9). The above results showed that all 18 variants in Table 1 were valid instrumental variables and could be used in the MR analysis.

**Table 2:** MR analysis using different methods for genetic associations between aspartate and prostate cancer^a^

MR method	Association					Heterogeneity	
	Estimate	SE^b^	95% CI (Down)	95% CI (Up)	*P*-value	Q	*P*-value
Simple median	0.038	0.030	-0.021	0.096	0.206		
Weighted median	0.032	0.023	-0.012	0.077	0.155		
Penalized weighted median	0.032	0.023	-0.012	0.077	0.155		
IVW	0.042	0.020	0.003	0.081	0.034	1.868	0.3932
Penalized IVW	0.042	0.020	0.003	0.081	0.034		
Robust IVW	0.041	0.013	0.015	0.068	0.002		
Penalized robust IVW	0.041	0.013	0.015	0.068	0.002		
MR-Egger	-0.042	0.097	-0.232	0.147	0.660		
MR-Egger intercept	0.106	0.118	-0.126	0.338	0.370		
Penalized MR-Egger	-0.042	0.097	-0.232	0.147	0.660		
Penalized MR-Egger intercept	0.106	0.118	-0.126	0.338	0.370		
Robust MR-Egger	-0.042	0.046	-0.131	0.047	0.354		
Robust MR-Egger intercept	0.106	0.068	-0.028	0.240	0.121		
Penalized robust MR-Egger	-0.042	0.046	-0.131	0.047	0.354		
Penalized robust MR-Egger intercept	0.106	0.068	-0.028	0.240	0.121		
Robust adjusted profile score	0.043				0.041		

^a ^Single nucleotide polymorphisms with genome-wide significance (*P* < 5 × 10^–8^) were used as genetic predictors

^b ^*SE* Standard Error

**Table 3:** MR analysis using different methods for genetic associations between aspartate and breast cancer^a^

MR method	Association					Heterogeneity	
	Estimate	SE^2^	95% CI (Down)	95% CI (Up)	*P*-value	Q	*P*-value
Simple median	0.030	0.020	-0.009	0.068	0.134		
Weighted median	0.031	0.016	-0.001	0.063	0.060		
Penalized weighted median	0.031	0.016	-0.001	0.063	0.060		
IVW	0.032	0.015	0.004	0.061	0.028	0.4410	0.8021
Penalized IVW	0.032	0.015	0.004	0.061	0.028		
Robust IVW	0.032	0.007	0.018	0.046	0.000		
Penalized robust IVW	0.032	0.007	0.018	0.046	0.000		
MR-Egger	0.015	0.070	-0.122	0.152	0.827		
MR-Egger intercept	0.021	0.086	-0.148	0.191	0.805		
Penalized MR-Egger	0.015	0.070	-0.122	0.152	0.827		
Penalized MR-Egger intercept	0.021	0.086	-0.148	0.191	0.805		
Robust MR-Egger	0.015	0.022	-0.029	0.059	0.496		
Robust MR-Egger intercept	0.021	0.033	-0.044	0.087	0.521		
Penalized robust MR-Egger	0.015	0.022	-0.029	0.059	0.496		
Penalized robust MR-Egger intercept	0.021	0.033	-0.044	0.087	0.521		
Robust adjusted profile score	0.032				0.036		

^a ^Single nucleotide polymorphisms with genome-wide significance (*P* < 5 × 10^–8^) were used as genetic predictors.

^b ^*SE* Standard Error

**Table 4:** MR analysis for genetic associations between glutamate and prostate and breast cancers^a^

Outcome	MR method	Association			
		Estimate	95% CI (Down)	95% CI (Down)	*P*-value
Prostate Cancer	IVW	0.029	-0.053	0.110	0.490
Prostate Cancer	Robust adjusted profile score	0.029			0.506
Breast Cancer	IVW	0.041	-0.020	0.102	0.186
Breast Cancer	Robust adjusted profile score	0.041			0.215

^a ^Single nucleotide polymorphisms with genome-wide significance (*P* < 5 × 10^–8^) were used as genetic predictors

Furthermore, there was no sign of heterogenetic effects between the genetic effects and risk of prostate and breast cancers in the Cochran’s Q statistics test (Tables 2, 3 and Tables S1, S2, S3 and S4) and It was identified that the IVW estimate was not biased by the influence of particular SNPs in the leave-one-out sensitivity analysis, based on SNPs with genome-wide significance and more SNPs with less stringent significance (Figs. S6, S7 and S8).

## Discussion

Balanced diet/nutrition intake constitutes a preventive strategy for cancer incidences that in turn may impede the development and progression of cancer. Amino acids, glutamine and aspartate in particular, are vital alternative nutrients for cellular energetics and biomass synthesis apart from glucose. With consistency of the implication of evolutionary biology theory, our finding suggested that aspartate, with the potential to affect the endocrine system [40], was a underlying risk factor for prostate and breast cancers.

To the best of our knowledge, it is the first MR study to examine the potential causal effects of glutamate and aspartate on prostate and breast cancers. Genetically instrumented glutamate and aspartate can remove potential confounding factors in observational studies and make a difference of the effects of these two dietary programs, which are correlated and co-occur. And at the same time it can also minimize the measurement error in nutrition studies from self-reported dietary consumption [21]. Furthermore, it is cost-efficient depending on large GWASs and case–control studies with extensive genotyping [41]. The samples for MR analysis were from two completely separate GWASs, one sample for genetic variants on exposures (glutamate and aspartate) [42] and the other sample for genetic variants on outcomes (prostate cancer [23] and breast cancer [22]), which means any correlation in the sample with the exposures is unlikely to be replicated in the sample with the clinical outcomes.

Despite above-claimed strength, this study has several limitations. First, our findings on serum aspartate were seemingly inconsistent with anti-cancer effect of aspartate in food, such as soy [14, 15, 43–45]. A possible explanation is that the effects of serum glutamate and aspartate reflected endogenous exposures that may distinguish with exogenous dietary exposures; but levels of serum glutamate and aspartate are likely affected by dietary consumption [46]. Second, MR requires the genetic instruments associated with the exposures and the genetic variants are no pleiotropy. There are no confounders in the causal association [47]. As a result, here only 1 SNP associated with glutamate met the requirements with genome-wide significance threshold (*P* < 5 × 10^−8^). Thus, the exposure glutamate was dropped in the sensitivity analysis, which might lead to the decreased reliability of the result without MR-Egger and WM analysis. Therefore, more less strongly associated SNPs were included for analysis. However, the inconsistencies between the results of IVW and MR Egger analyses could also be caused by weak instrument bias and the potential differences in validities of all the selected SNPs. Third, the genetic associations in our study were from studies largely conducted in European descent with genomic control [21, 48]. Some genetic variants may be different from other populations, which was caused by “population bottlenecks” [49]. Thus, the results in our study might not be applied to other populations in other parts of the world, although the allele frequency of major SNP was similar in ethnic groups. GWAS datasets from other populations should be collected to replicate and confirm the findings. Fourth, it could not be assessed whether in our estimates the effects of glutamate and aspartate on cancers vary by sex or age. The stratified MR analysis should be performed. Fifth, to reduce the possibility of false positive results, a Bonferroni correction (corrected *P*: 0.05/4 = 0.0125) of multiple independent tests (tests for associations of two metabolites with two types of cancer, respectively) should be used. Therefore, our findings were deemed suggestive evidence of possible associations (0.0125 < *P* < 0.05). Hence, this necessitates further studies to replicate our findings and get more conclusive results. Last but not the least, the underlying pathways of the causal effects remained to be clarified.

Aspartate and glutamate belong to the arginine family, along with asparagine, glutamine and arginine itself. They are inter-convertible via complex metabolism in most mammals. In our findings, aspartate was the risk factor for prostate cancer and breast cancer development. Emerging evidence reveals that glutamine and interlinked asparagine metabolism may be critical for endothelial cell (EC) metabolism, as a regulator of angiogenesis [50]. Therefore, the fact that the serum levels of aspartate and glutamine serving as a risk factor might be exerted via their relevant metabolites given that asparagine and glutamine are known to promote cancer cell proliferation and vessel sprouting. Furthermore, in one breast cancer model, asparagine bioavailability impacts the ratios of epithelial-to-mesenchymal-like tumor cells and tumor progression [51]. In the epithelial‐mesenchymal transition (EMT) and PCa progression, aspartate is generally a recognized contributor, its metabolism when elevated is accompanied with high levels of adenylosuccinate, arginosuccinate, malate, asparagine known to be correlated with tumor progression [52].

Another plausible mechanism underlying our findings is a link between aspartate and arginine via the urea cycle. The urea cycle detoxifies free ammonia in the livers of mammals, in which arginine is synthesized in two steps: citrulline and aspartate are used to synthesize argininosuccinate which is then converted to arginine. Arginine is a non-essential amino acid in adults but is necessary for fast-growing cells such as cancer cells. Currently Graboa et al. [53] have reported that arginine is crucial during malignancy development. Arginine deprivation has been a novel and promising approach to treat tumors that are not hepatocyte-derived thus unable to self-suffice for arginine owing to a lack of the urea cycle [54]. However, the effects of glutamate and aspartate on human health are very complex. Some studies show that nutritional supplements, aspartate and glutamate, possess beneficial health and anti-oxidative effects. For example, aspartate can improve liver metabolism [55], and glutamate can modulate the body weight [56], regulate the release of hormones [57] and lipid metabolism [58], probably owing to its impact upon the TCA cycle and ATP production [59]. Aspartate might also operate by lowering androgens [10], and high level of circulating androgens is a risk factor for prostate cancer, a notion for which there is, however limited, evidence in human studies [16].

Currently, the technique of ultra-high performance liquid chromatography-tandem mass spectrometry measuring levels of amino acids such as aspartate and glutamate has been well validated [60]. It will be worthwhile to exploit more relevant genetic instruments if available. Our work, based on MR studies that constitute a tool for testing causation, cannot dictate the exact size/degree of causal effects [61] nor can replace clinical trials; however, the findings built on the ever-growing knowledge about the effects of glutamine and aspartate on prostate cancer and breast cancer development is for sure greatly relevant to dietary recommendations, along with providing guidance for cancer prevention as well as public health in general.

## Supplementary Information

**Additional file 1: Fig. S1.** Forest plot of the causal effects of aspartate (3 independent SNPs, *P* value <5×10^−8^) on prostate and breast cancers. IVW, inverse-variance weighted. (a). The association of aspartate with prostate cancer. (b). The associationof aspartate with breast cancer.

**Additional file 2: Fig. S2.** Causal associations between aspartate and prostate and breast cancers.

**Additional file 3: Fig. S3.** Causal associations between glutamate and prostate and breast cancers.

**Additional file 4: Fig. S4.** Forest plot of the causal effects of aspartate (13 independent SNPs, *P* value <5×10^−6^) on prostate and breast cancers. IVW, inverse-variance weighted. (a). The association of aspartate with prostate cancer. (b). The associationof aspartate with breast cancer.

**Additional file 5: Fig. S5.** Forest plot of the causal effects of glutamate (5 independent SNPs, *P* value < 5×10^−6^) on prostate and breast cancers. IVW, inverse-variance weighted. (a). The association of glutamate with prostate cancer. (b). The association of glutamate with breast cancer.

**Additional file 6: Fig. S6.** Leave-one-out sensitivity analysis for the effect of aspartate (3 independent SNPs with *P *value < 5×10^−8^) on prostate and breast cancers. IVW, inverse-variance weighted. (a). The association of aspartate with prostate cancer. (b). The association of aspartate with breast cancer.

**Additional file 7: Fig. S7.** Leave-one-out sensitivity analysis for the effect of aspartate (13 independent SNPs with *P *value < 5×10^−6^) on prostate and breast cancers. IVW, inverse-variance weighted. (a). The association of aspartate with prostate cancer. (b). The association of aspartate with breast cancer.

**Additional file 8: Fig. S8.** Leave-one-out sensitivity analysis for the effect of glutamate (5 independent SNPs with *P* value < 5×10^−6^) on prostate and breast cancers. IVW, inverse-variance weighted. (a). The association of glutamate with prostate cancer. (b). The association of glutamate with breast cancer.

**Additional file 9: Fig. S9.** Single MR estimates from each of the genetic variants using MR-Egger method. The black scatter plots indicate single causal estimates from each of the genetic variants associated with serum aspartate or glutamate level on the x-axis and prostate cancer or breast cancer on the y-axis. The continuous line represents the causal effect of serum aspartate or glutamate level on prostate cancer or breast cancer. (a-b). The estimates between aspartate and cancers using 3 independent SNPs with *P* value < 5×10^−8^, (a). Prostate cancer; (b). Breast cancer. (c-d). The estimates between aspartate and cancers using 13 independent SNPs with *P* value < 5×10^−6^, (c). Prostate cancer; (d). Breast cancer. (e-f). The estimates between glutamate and cancers using 5 independent SNPs with *P* value < 5×10^−6^, (e). Prostate cancer; (f). Breast cancer.

**Additional file 10: Table S1.** MR analysis using different methods for genetic associations between aspartate and prostate cancer^1^.

**Additional file 11: Table S2.** MR analysis using different methods for genetic associations between aspartate and breast cancer^1^.

**Additional file 12: Table S3.** MR analysis using different methods for genetic associations between glutamate and prostate cancer^1^.

**Additional file 13: Table S4.** MR analysis using different methods for genetic associations between glutamate and breast cancer^1^.

**Additional file 14: Table S5.** The characteristics of genome-wide association studies on the included outcomes.

## Data Availability

All data analyzed in this study are included in the manuscript and supplementary materials.
