# Supplementary figures and images for: Effects of glutamate and aspartate on prostate cancer and breast cancer: a Mendelian randomization study
Source: BMC Genomics. 2022 Mar 16;23:213. doi: 10.1186/s12864-022-08442-7 (PMC8925075; doi:10.1186/s12864-022-08442-7)

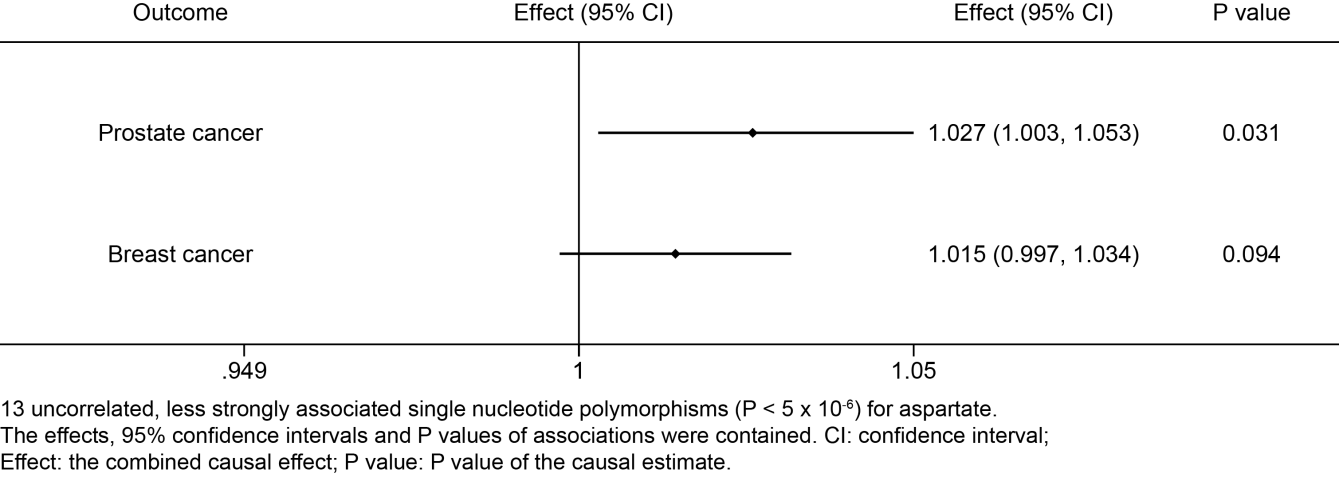


Fig. S2. Causal associations between aspartate and prostate and breast cancers.

Supplement: Supplementary file 2 — Additional file 2: Fig. S2. Causal associations between aspartate and prostate and breast cancers. [file 12864_2022_8442_MOESM2_ESM.docx]

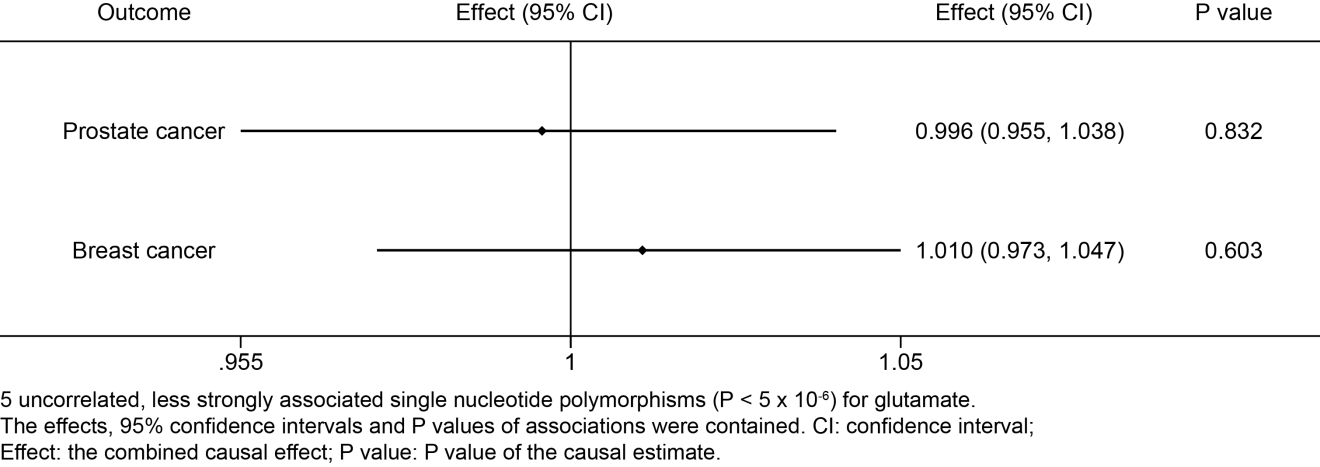


Fig. S3. Causal associations between glutamate and prostate and breast cancers.

Supplement: Supplementary file 3 — Additional file 3: Fig. S3. Causal associations between glutamate and prostate and breast cancers. [file 12864_2022_8442_MOESM3_ESM.docx]

Table S5. The characteristics of genome-wide association studies on the included outcomes


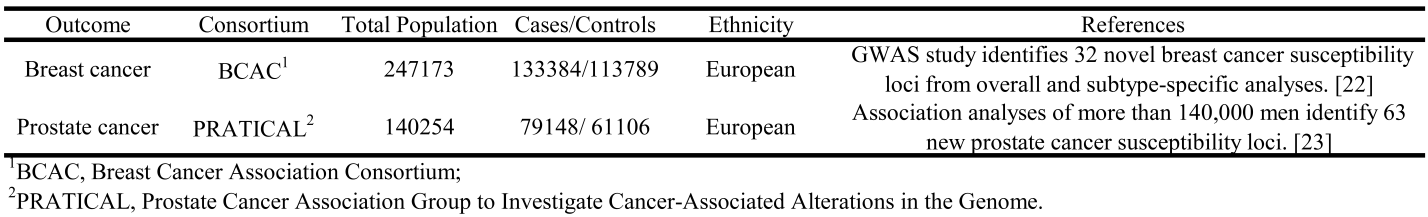

Supplement: Supplementary file 14 — Additional file 14: Table S5. The characteristics of genome-wide association studies on the included outcomes. [file 12864_2022_8442_MOESM14_ESM.docx]
