# Supplementary material for: Effects of glutamate and aspartate on prostate cancer and breast cancer: a Mendelian randomization study
Source: BMC Genomics. 2022 Mar 16;23:213. doi: 10.1186/s12864-022-08442-7 (PMC8925075; doi:10.1186/s12864-022-08442-7)
Supplement: Supplementary file 5 — Additional file 5: Fig. S5. Forest plot of the causal effects of glutamate (5 independent SNPs, P value < 5×10^−6^) on prostate and breast cancers. IVW, inverse-variance weighted. (a). The association of glutamate with prostate cancer. (b). The association of glutamate with breast cancer. [file 12864_2022_8442_MOESM5_ESM.docx]

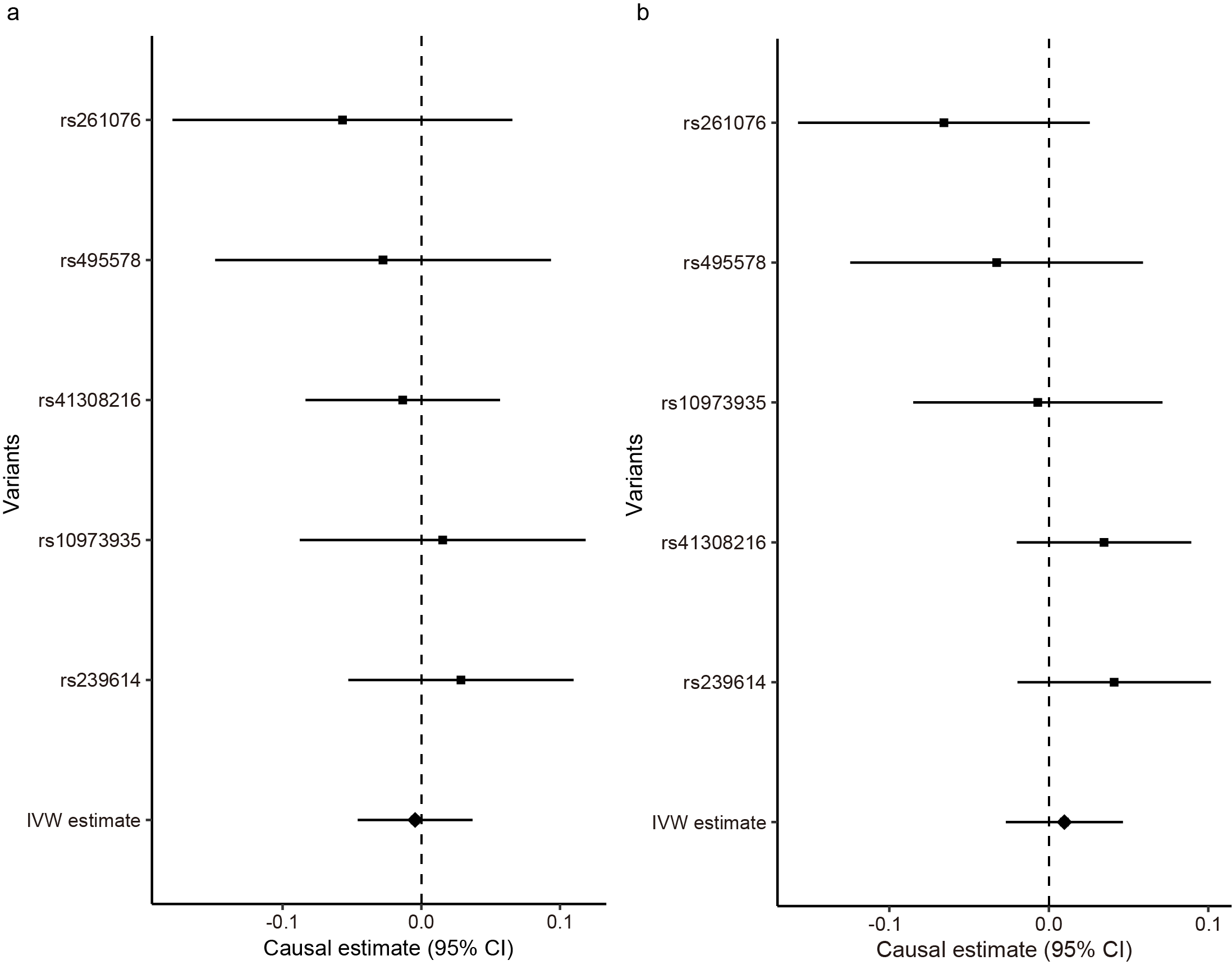


Fig. S5 Forest plot of the causal effects of glutamate (5 independent SNPs, P value < 5×10^−6^) on prostate and breast cancers. IVW, inverse-variance weighted. (a). The association of glutamate with prostate cancer. (b). The association of glutamate with breast cancer.
