# Supplementary material for: Effects of glutamate and aspartate on prostate cancer and breast cancer: a Mendelian randomization study
Source: BMC Genomics. 2022 Mar 16;23:213. doi: 10.1186/s12864-022-08442-7 (PMC8925075; doi:10.1186/s12864-022-08442-7)
Supplement: Supplementary file 7 — Additional file 7: Fig. S7. Leave-one-out sensitivity analysis for the effect of aspartate (13 independent SNPs with P value < 5×10^−6^) on prostate and breast cancers. IVW, inverse-variance weighted. (a). The association of aspartate with prostate cancer. (b). The association of aspartate with breast cancer. [file 12864_2022_8442_MOESM7_ESM.docx]

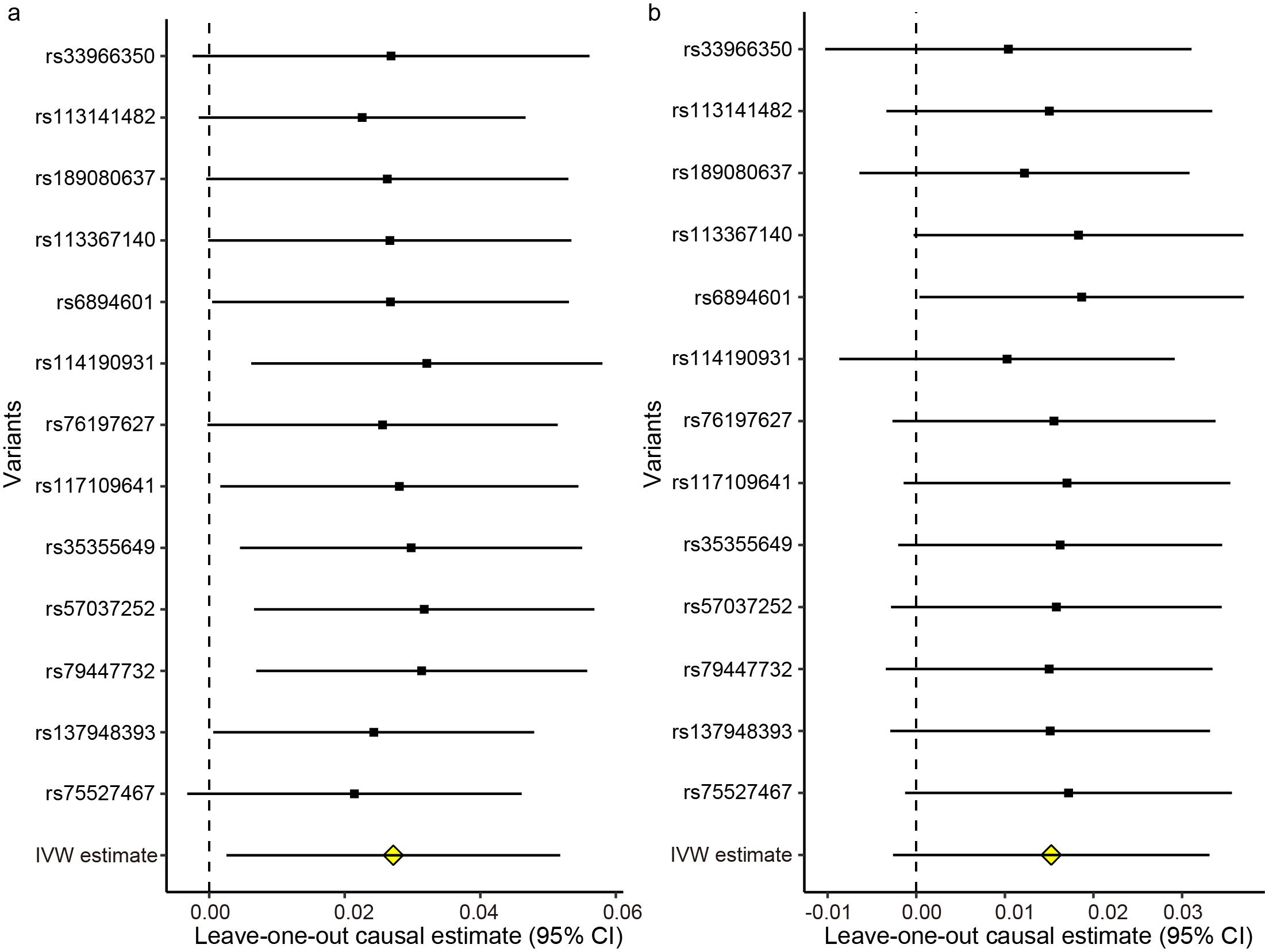


Fig. S7 Leave-one-out sensitivity analysis for the effect of aspartate (13 independent SNPs with P value < 5×10^−6^) on prostate and breast cancers. IVW, inverse-variance weighted. (a). The association of aspartate with prostate cancer. (b). The association of aspartate with breast cancer.
