# Supplementary material for: Effects of glutamate and aspartate on prostate cancer and breast cancer: a Mendelian randomization study
Source: BMC Genomics. 2022 Mar 16;23:213. doi: 10.1186/s12864-022-08442-7 (PMC8925075; doi:10.1186/s12864-022-08442-7)
Supplement: Supplementary file 9 — Additional file 9: Fig. S9. Single MR estimates from each of the genetic variants using MR-Egger method. The black scatter plots indicate single causal estimates from each of the genetic variants associated with serum aspartate or glutamate level on the x-axis and prostate cancer or breast cancer on the y-axis. The continuous line represents the causal effect of serum aspartate or glutamate level on prostate cancer or breast cancer. (a-b). The estimates between aspartate and cancers using 3 independent SNPs with P value < 5×10^−8^, (a). Prostate cancer; (b). Breast cancer. (c-d). The estimates between aspartate and cancers using 13 independent SNPs with P value < 5×10^−6^, (c). Prostate cancer; (d). Breast cancer. (e-f). The estimates between glutamate and cancers using 5 independent SNPs with P value < 5×10^−6^, (e). Prostate cancer; (f). Breast cancer. [file 12864_2022_8442_MOESM9_ESM.docx]

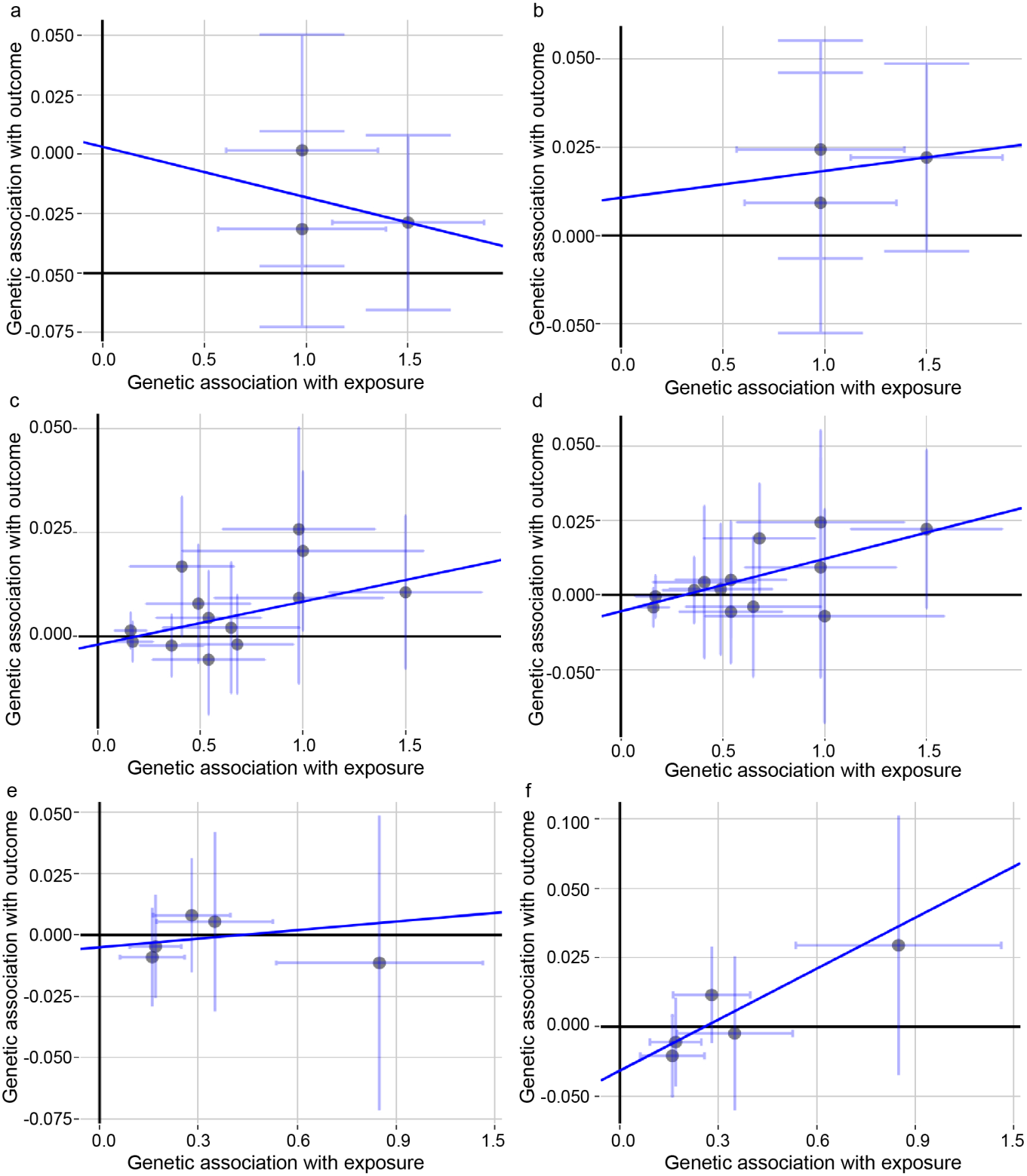


Fig. S9 Single MR estimates from each of the genetic variants using MR-Egger method. The black scatter plots indicate single causal estimates from each of the genetic variants associated with serum aspartate or glutamate level on the x-axis and prostate cancer or breast cancer on the y-axis. The continuous line represents the causal effect of serum aspartate or glutamate level on prostate cancer or breast cancer. (a-b). The estimates between aspartate and cancers using 3 independent SNPs with P value < 5×10^−8^, (a). Prostate cancer; (b). Breast cancer. (c-d). The estimates between aspartate and cancers using 13 independent SNPs with P value < 5×10^−6^, (c). Prostate cancer; (d). Breast cancer. (e-f). The estimates between glutamate and cancers using 5 independent SNPs with P value < 5×10^−6^, (e). Prostate cancer; (f). Breast cancer.
