# Supplementary material for: Effects of glutamate and aspartate on prostate cancer and breast cancer: a Mendelian randomization study
Source: BMC Genomics. 2022 Mar 16;23:213. doi: 10.1186/s12864-022-08442-7 (PMC8925075; doi:10.1186/s12864-022-08442-7)

Table S2. MR analysis using different methods for genetic associations between aspartate and breast cancer^1^


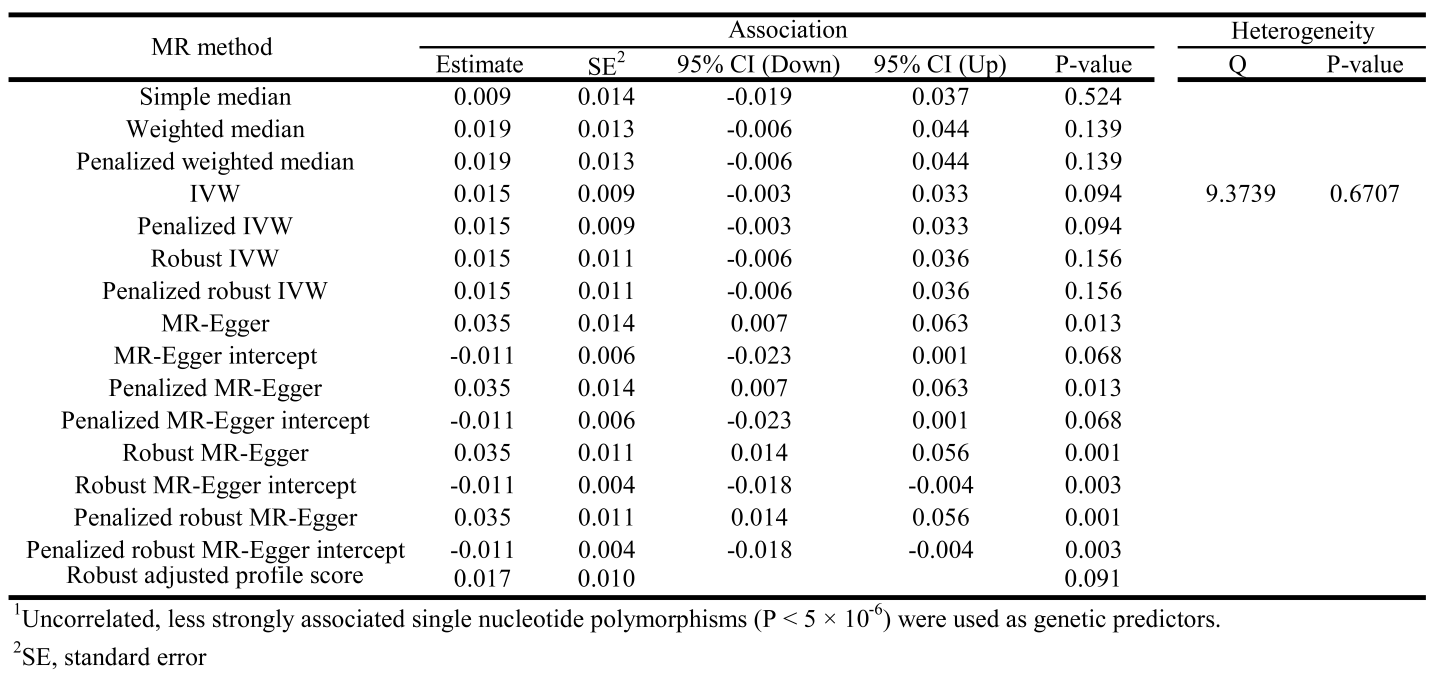

Supplement: Supplementary file 11 — Additional file 11: Table S2. MR analysis using different methods for genetic associations between aspartate and breast cancer1. [file 12864_2022_8442_MOESM11_ESM.docx]
